# What Motivates Intensive Care Nurses to Continue Working in the Intensive Care Unit: A Qualitative Study

**DOI:** 10.1177/23333936251349352

**Published:** 2025-06-17

**Authors:** Camilla Leithe-Lajord, Kjersti Grønning

**Affiliations:** 1 Nord-Trøndelag Hospital Trust, Levanger Hospital, Norway; 2 Norwegian University of Science and Technology, Trondheim, Norway

**Keywords:** nurse retention, intensive care nurses, motivation, work environment, professional focus, professional development, leadership, engagement, Norway

## Abstract

The COVID-19 pandemic highlighted the need to improve ICU capacity and working conditions in Norwegian hospitals and recruit and retain nurses. The aim of this study is to explore what motivates nurses to continue working in the ICU using a constructivist approach, asserting that knowledge is created through the interaction between the researcher, the participants, and the context. Eight individual semi-structured interviews with intensive care nurses were audio-recorded and transcribed verbatim. The data were analysed using thematic analysis. We identified one main overarching theme, “an interpersonal and professionally focussed work environment,” highlighting how a supportive and professional atmosphere boosts intensive care nurses’ job satisfaction and motivation, and three subthemes. The sub-themes were named: “unity and well-being,” underscoring the need for professional and interpersonal support, “close and professional leadership,” emphasising the importance of having an attentive, accessible, and unifying leader, and “professional engagement and mastery,” focussing on the significance of training, continuous skill development, and task distribution based on competence and experience. This study adds new knowledge about environmental factors that contribute to the understanding of why intensive care nurses remain in their profession and their motivation to continue working in the ICU despite the high workload.

## Introduction

Nurses comprise more than half of all healthcare professionals and play a crucial role in the healthcare sector. Since the onset of the COVID-19 pandemic in 2020, the essential role of nurses in society and healthcare systems worldwide has become more apparent than ever (Liu et al., 2020). When the pandemic reached Norway, attention quickly turned to the preparedness and working conditions in intensive care units (ICUs) in Norwegian hospitals (Lie et al., 2021) and revealed a shortage of intensive care nurses (Christensen & Lægreid, 2023). The COVID-19 pandemic also increased existing challenges related to work-life balance for professionals with inflexible and long working hours. This led to increased stress, emotional burden and reduced quality of life, and underscores the urgent need for a comprehensive approach that addresses both the emotional and physical demands of nursing in general (Antolí-Jover et al., 2024).

Research shows that nurses are driven to excel in their profession when the organisation clearly outlines roles, goals, and tasks. This clarity allows nurses to influence routines and work schedules, practice nursing with a humanistic approach, and promote effective teamwork among skilled, qualified, and supportive colleagues (Hörberg et al., 2023). It is also found that the nature of work in ICUs itself contributes significantly to nurses’ job satisfaction (Teruya et al., 2019), along with workplaces focussing on collegial support, available resources, and opportunities for professional competence development (Spence Laschinger & Fida, 2015). It is shown that motivation to work as a nurse or intention to leave the profession is a complex phenomenon influenced by various factors (Hörberg et al., 2023). A recent scoping review highlighted that autonomy, team composition, and professional relationships were important in shaping job satisfaction among nurses (Vleminckx et al., 2024). Researchers have also reported that a motivating and supportive work environment with a clear, inclusive, and authentic leader is crucial for retaining intensive care nurses in their jobs (Al Yahyaei et al., 2022; Spence Laschinger & Fida, 2015). Nurses also find their jobs more motivating if they receive sufficient pay that reflects the actual workload they perform (Abu Yahya et al., 2019), and when the work environment is embodied by good collaboration, effective communication, a patient-centred culture (Al Sabei et al., 2022; Sawatzky et al., 2015), and opportunities for competence development through interaction and cooperation (Høgbakk & Jakobsen, 2019).

On the other hand, studies indicate that burnout among nurses is linked to job stress (Chen et al., 2025; Dilig-Ruiz et al., 2018) and that dysfunctional work environments have detrimental effects on both patient care and nurse retention (Ahmed et al., 2024; Kiptulon et al., 2024). Heavy workload (Cooper, 2024; Yanbei et al., 2023), insufficient staffing, high-stress environment of ICUs (Lima et al., 2023; Yanbei et al., 2023) and demands of caring for critically ill patients are also major contributing factors to burnout among intensive care nurses. (Cooper, 2024). In one study, the researchers concluded that implementing flexible labour policies and psychological support programmes could reduce job stress and prevent burnout (Antolí-Jover et al., 2024).

Even though several studies show that a positive work environment and organisational cultures are important and beneficial for job satisfaction among intensive care nurses (Kiptulon et al., 2024; Salehi et al., 2020; Tzenalis et al., 2023; Yanbei et al., 2023), there are concerns at both national (Norwegian Ministry of Health and Care Services, 2023) and international levels (Lasater, 2024) about how to ensure an adequate supply of intensive care nurses in the future. Therefore, this study aims to explore what motivates nurses to continue working in the ICU.

## Methods

To explore what motivates nurses to continue working in the ICU, we adopted an exploratory descriptive approach. This approach is well suited to explore and describe the experiences of intensive care nurses about factors that motivate them to continue working in the ICU (Hunter et al., 2019; Renjith et al., 2021; Sandelowski, 2000). The study is conducted within a constructivist paradigm, which posits that knowledge is formed through the interaction between the researcher, the participants, and the context (Braun & Clarke, 2021). Our orientation is that the interpretations should stay close to the data, being recognisable to the participants. The data were collected through individual semi-structured interviews with intensive care nurses currently working in the ICU. By using semi-structured interviews, we can ensure flexibility in the conversation, enabling the participants to express their thoughts freely while still covering key topics relevant to the study (Braun & Clarke, 2021).

### Study Setting

The study was conducted at an ICU in a local hospital in Mid-Norway serving about 100,000 inhabitants in the southern part of Nord-Trøndelag. The ICU provides medical treatment and nursing care to severely/critically ill patients after major surgeries, serious accidents, or other illnesses. It consists of seven intensive care rooms and two monitoring rooms. The nurse-patient ratio is 1:1. There are usually 10 nurses during the day shift, 6 in the evening, and 4 on the night shift. All nurses are specialised in intensive care nursing, and the staff retention rate is relatively high. To ensure sufficient staffing, nurses may apply for educational positions to become intensive care nurses paid by the hospital. The ICU also has a union representative for the intensive care nurses and a safety delegate. The safety delegate’s task is to safeguard all employees’ interests in matters concerning the work environment, ensuring that the hospital takes care of the employees’ safety, health, and welfare and that the Working Environment Act is followed.

### Recruitment and Data Collection

A purposive sampling approach was used to recruit intensive care nurses for this study. The first author attended a physical meeting at the ICU to inform the nurses about the study, in addition to emailing an invitation letter with information about the purpose of the study. The invitation letter also contained information about the researchers’ background and that participation in the study was voluntary. Eight out of 45 intensive care nurses working at the ICU responded to the invitation email. The time and place for the interviews were decided individually. The leadership at the ICU provided an appropriate room at the hospital for the interviews and allowed the nurses to participate during their working hours. The nurses provided written consent to participate in the study prior to the interviews.

The first author developed a thematic interview guide in collaboration with the second author, containing two introductory questions about the intensive care nurses’ work experience and why they wanted to become intensive care nurses. The main questions assessed how the nurses in general experienced working as an intensive care nurse, how a typical working day was, what the nurses found as positive about their job, challenges in their job, what has been crucial for working in the ICU, what has been important enjoyment at work, and if the nurses had experienced periods of low motivation or having thoughts about leaving the profession. The questions were open-ended with follow-up probing questions to elaborate on their answers. The interviews were completed between February and March 2023. The interviews were conducted by the first author, who has a professional background as nurse and is a female Norwegian novice researcher. The interviews lasted between 30 and 60 min. Notes were taken during the interviews, and audio recordings were made to ensure no valuable information was lost. The audio recordings were transcribed verbatim by the first author.

### Data Analysis

The interviews were analysed using thematic analysis (Braun & Clarke, 2021) because this method is suitable for a constructivist perspective on knowledge. The epistemological foundation of thematic analysis is that knowledge is socially constructed, representing a co-creation between the researcher and the participants/data, and it acknowledges that data can have multiple meanings, allowing for interpretation (Graneheim et al., 2017). Thematic analyses enable a detailed and nuanced understanding of the nurses’ experiences and perspectives, providing rich qualitative data that can uncover insights into the complexities of their work environment and practices. Thematic analysis is also a theoretically flexible approach that accommodates various ontologies, epistemologies, and research questions (Nowell et al., 2017), highlighting reflexivity as a key aspect of the method (Braun & Clarke, 2021).

During the first phase of the analysis, it was important to become thoroughly familiar with the collected data material (Braun & Clarke, 2021). The first author transcribed all interviews verbatim in their entirety. Notes were taken along the way to highlight important paragraphs, sentences, or opinions. The process of creating initial codes for the data material then commenced, involving identifying relevant aspects of the transcriptions and categorising them into codes based on identified themes or patterns. An example of the coding process is shown in Table 1 below.

**Table 1.** Example of the Coding Process.

Data excerpt	Coded to
“That is, I still think it is a really interesting job, and I look forward to going to work every day. So, there couldn’t be any better place for me to work, but of course. Since I have an education in Intensive Care Nursing, then maybe it’s not so interesting working in any other unit.”	Very interesting job
	Looking forward to going to work
	Can’t think of a better place to work
	Have education in Intensive Care Nursing, not relevant working anywhere else than in an Intensive Care Unit

Quotes, paragraphs, and sentences were sorted, resulting in the creation of multiple codes such as “positive work environment,” “varied tasks”, and “exciting workday.” Text excerpts, quotes, and paragraphs were not limited to a single code but could be assigned to several codes during this phase of the analysis. The next phase focussed on constructing themes (Braun & Clarke, 2021). This involved organising the identified codes into broader themes or categories. These themes aimed to uncover connections within the data material and align with the research question. An example of the analysing process is presented in Table 2 below.

**Table 2.** Example of Sorting Codes into Themes.

Focus on the profession	Motivation	Reasons for remaining in the ICU
- Inherent interest for the profession and professional development	- Caring for intensive care patients	- Interdisciplinary environment
	- To see that your job makes a difference	- Varied work tasks
	- Feeling of mastery	- Professional development
- Engaging and professionally oriented work environment	- To implement the newest knowledge and research in their work	- Exciting patient group
- Quality assurance	- Being able to develop professionally and personally	
- Given time to practice and simulate to ensure skills development		

By the end of this phase, a preliminary draft of the overarching theme, along with the associated subthemes and codes, was prepared for the final description of the findings. In the subsequent phase, both researchers critically reviewed the themes, discussing whether they addressed the study’s purpose and research question. Themes and categories were reviewed and reorganised, with some themes being merged and others being divided (Braun & Clarke, 2021). Finally, the main theme with corresponding subthemes were defined. The analysis was conducted in Norwegian, but the main text, themes, subthemes, and quotes were translated into English during phase 6.

### Ethical Considerations

All participants received oral and written information about the purpose of the study, that participation in the project was voluntary, and that the data would be collected through audio-recorded individual interviews. The audio recordings were deleted after the transcriptions were checked and re-checked for accuracy. The data material containing the participants’ personal information was anonymised and no personal data was linked to the transcripts. The study was approved by the Norwegian Agency for Shared Services in Education and Research (ref. 904670) and the Data Access Committee (DAC) of the relevant health institution (ref. 2023/33-1531/2023) before commencement.

### Trustworthiness

The study was guided by recognised criteria for trustworthiness, credibility, transferability, dependability, and confirmability (Lincoln & Guba, 1985). Credibility was enhanced by the knowledge and experience the researchers had from the research field, the working environment for nurses. It made us confident about the relevance of the research question and that the population of intensive care nurses were suitable to inform the research question. Since both authors are nurses, it is crucial to recognise our subjectivity and acknowledge that our nursing background may have influenced the interviews and analysis (Braun & Clarke, 2021). Reflexivity, however, is an ongoing process of reflection throughout the entire research process and is never fully complete. The interpretation of the data occurs at all stages, as it involves the researchers’ subjectivity, and the decisions made before and during data collection, and throughout all phases of analysis. To ensure credibility, we also discussed our preliminary findings with other researchers and solicited their feedback and comments to further refine our analysis process. The constructivist approach required us to be mindful of our roles, prior knowledge, assumptions, and values, and how these factors influenced the research. We provided a detailed description of the method, enabling a clear trace from data collection to analysis and, ultimately, the conclusion. Additionally, we included direct excerpts from participants to verify and highlight the key findings and interpretations within the themes (Braun & Clarke, 2021). Transferability, referring to the applicability of the findings to other contexts or groups (Lincoln & Guba, 2016) is ensured by providing information and description about context, data collection, and the process of analysis, making it possible for the readers to decide for themselves whether the results are transferable to their context, the ICU setting. Transferability and authenticity were further bolstered by providing detailed descriptions of the participants and the context of their experiences, and by using direct quotes from their responses. Dependability pertains to the consistency and stability of the research project over time (Lincoln & Guba, 2016; Polit & Beck, 2021). In this study, we maintained a stable team of researchers where the same researcher conducted the interviews, transcribed, and coded the data. The research team worked collaboratively to interpret the data, develop themes, and finalise the results.

## Findings

The sample consisted of three male and five female intensive care nurses, all of whom were of ethnic Norwegian origin, with work experience as intensive care nurses ranging from 4 to 31 years. The participants’ demographics is presented in Table 3 below.

**Table 3.** Participants’ Demographics.

Participant	Gender	Years of ICU experience
Intensive Care Nurse 1	Female	31
Intensive Care Nurse 2	Male	31
Intensive Care Nurse 3	Female	13
Intensive Care Nurse 4	Female	5
Intensive Care Nurse 5	Male	19
Intensive Care Nurse 6	Male	13
Intensive Care Nurse 7	Female	4
Intensive Care Nurse 8	Female	14

We identified one main overarching theme, and three subthemes as presented in Table 4 below.

**Table 4.** Main Theme with Corresponding Subthemes.

An interpersonal and professionally focussed work environment		
Unity and well-being	Close and professional leadership	Professional engagement and mastery

The main theme, “an interpersonal and professionally focussed work environment,” highlights how a supportive and professional atmosphere boosts intensive care nurses’ job satisfaction and motivation. The first subtheme, “unity and well-being,” underscores the need for professional and interpersonal support. The second subtheme, “close and professional leadership,” emphasises the importance of having an attentive, accessible, and unifying leader, and the third subtheme, “professional engagement and mastery,” focuses on the significance of training, continuous skill development, and task distribution based on competence and experience.

The themes are further elaborated with rich descriptions and illustrated with direct quotes from the participants below. To ensure the anonymity of the participants, the quotes are labelled with their respective participant numbers.

### An Interpersonal and Professionally Focussed Work Environment

The participants highlighted that a work environment focussed on professionalism and inclusivity not only fostered motivation but also played a crucial role in their decision to stay in the ICU. They further underlined that their work environment was characterised by mutual respect and clear communication, sharing of knowledge, fair distribution of patients and tasks, and assignment of responsibilities based on competence and capacity to provide high-quality care to their patients. Everyone collaborated to create a culture where the nurses felt valued, respected, and had equal opportunities.

When I look around, I recognize the value of my workplace and my colleagues. My colleagues, in particular, have been crucial for me thinking “I can’t quit this place, it is so good here.” And then it isn’t the profession in itself, it’s the actual work environment. (Intensive Care Nurse 7)

When the participants were asked about what motivated them in their daily work, they emphasised the ICU’s focus on professional development and quality of patient care.

In a good work environment, you get to develop both socially and professionally (. . .) You need to want that person lying in the bed to get better. Whether it involves ensuring the patient has a dignified end of life or treating them to ensure they survive, the intention must be there. (Intensive Care Nurse 5)

The intensive care nurses further explained that they had regular professional updating days as part of the rotation. One of the nurses also had a special responsibility for the professional development and quality of care in the ICU. The participants highlighted that they valued both the practical scenario training and theoretical days, organised by this intensive care nurse in collaboration with the management and collaborating units in the hospital. “It’s all about facilitating your ability to evolve professionally, in the work rotation for example. . . Having days set aside for theoretical instruction, courses and things like that” (Intensive Care Nurse 2). This professional and supportive atmosphere played a crucial role in their decision to continue working in the ICU, as one of the participants said: “Yes, when those who want responsibility get responsibility, you’ll take more ownership of your workplace, and then you care more” (Intensive Care Nurse 5).

The participants also stressed the importance of ensuring a fair distribution of patients and tasks within the workforce to avoid unnecessary dissatisfaction and demotivation.

So, if you come to work and feel like you have to do the same dull tasks every time, when everyone else has it so easy. Why do they get the most interesting patient cases (. . .) I am very conscious about making it as fair as possible (. . .) not to benefit anyone and treating everyone the same. (Intensive Care Nurse 5)

Similarly, they emphasised that workload should be managed to prevent wear and tear on individuals, and that no one should be assigned responsibilities beyond their competence and capacity. Several of the participants mentioned that they created their own identity and motivation around being an intensive care nurse. This deep connection to their profession not only maintained their dedication but also enhanced their overall work experience. One of the participants said it like this: “That's who I am, and that's what I can do!” (Intensive Care Nurse 1).

### Unity and Well-Being

Several participants said that the positive work environment, characterised by a strong unity and overall well-being, had developed gradually and significantly influenced their motivation to remain in the ICU. They highlighted the importance of a workplace where everyone is seen and valued, where there is space to discuss or address concerns, and where recognition and acknowledgement for their efforts come from both colleagues and the immediate leader. This supportive atmosphere fosters a sense of belonging and professional satisfaction, making it a crucial factor in their continued commitment to their roles in the ICU.

I think it’s a cultural thing for us. It is something we have worked up in the ICU and many of us are conscious about keeping it this way. That goes for both the social and the professional part. If the professional competency hadn’t been good, we wouldn’t be able to joke around with each other. In my view, it’s all interconnected. (Intensive Care Nurse 6)

They highlighted also the significance of looking out for one another, acknowledging each other, and providing support both professionally and personally. One of the participants expressed it like this:

There is an incredibly positive work environment here. For someone like me, who has always preferred to keep work and private life separate, it’s become challenging because the people are so pleasant. I have been a bit surprised by how significant it is. Having a good time at work means a lot. (Intensive Care Nurse 7)

The participants further put emphasis on sharing experiences with each other to provide the best possible care for the patient. They recognised that they had different strengths across the team and respected each other’s differences. There was always someone to discuss various patient situations with, and the participants felt there was a high level of openness to discuss challenging situations. This created space for regular debriefing without it necessarily being planned or organised.

For the most part, we are all professionally engaged and up-to-date. We enjoy collaborating and sharing experiences, supporting each other. Each of us has our areas of expertise, and we exchange knowledge, benefiting from each other’s strengths. . . We continue to grow from this foundation, creating an engaging and professional environment. (Intensive Care Nurse 3)

### Close and Professional Leadership

The participants highlighted the nature of the relationships between the nurses and leaders as important, and said that a visible and clear leader in the ICU was essential for the work environment. They emphasised the significance of a leader who took a comprehensive professional responsibility and collaborated with the team to drive the field of intensive care nursing forward. They valued a leader who was attentive to their personal and professional needs, easily accessible, and capable of uniting the ICU into a cohesive team: “That you worked closely with the management (. . .) and the section leader (. . .), that the management is attentive and has ambitions and ideas, that is important. Yes, leadership is the alpha and omega, I would say.” (Intensive Care Nurse 1).

Being involved in decisions and having the opportunity to influence their workday was perceived as important, rewarding, and motivating. The participants mentioned that they had regular meetings in the ICU where they could discuss what concerned them in their daily work. They also pointed out that if decisions were made without their input or sufficient information, it was demotivating and could create dissatisfaction.

I think the worst thing for many here is when decisions are made from above without them being informed, and then not being heard. That creates resistance, and then you have a workforce that is not satisfied. The intensive care unit has been quite good over the last 10 years, not necessarily due to a success formula, but because everyone has a voice. (Intensive Care Nurse 5)

Another point raised by the participants was their appreciation for the leadership’s efforts to maintain adequate staffing levels. Adequate staffing gave them a sense of security that they had sufficient competence and resources to provide safe and high-quality intensive care. In addition to this, during evening and night shifts, the intensive care nurse in charge of overseeing the ICU would call in extra nursing personnel if needed because of patient occupancy:

When I’m at work and in charge of the ICU, we must always operate responsibly. Even if my leader says everything is fine, if I see issues with staffing or our tasks, we need to call in more people, then we do so. (Intensive Care Nurse 8)

Moreover, the participants highlighted the importance of being valued in their work through good salary conditions. “And then there’s salary, of course. It has gotten better gradually (. . .) It is what we live off, basically” (Intensive Care Nurse 2). They believed that good salary conditions are crucial for recruiting and retaining intensive care nurses, as working shifts, including weekends and holidays, is not seen as attractive, although some also found it advantageous to work shifts. “I like working shifts, I wouldn’t want to work only during the daytime. I desire working more nights, it works well when you have small children” (Intensive Care Nurse 4). They further discussed that the framework conditions around the profession need to be strengthened, including ensuring salaries that reflect work effort and workload, as well as good senior policies, with days off, or reduced working hours to retain experienced intensive care nurses longer.

Maybe a bit more, maybe one day off, I’m just throwing it out there, maybe one day a week off with the same salary, a bit reduced working hours for the same salary, for example. Perhaps someone would remain in their profession for a few more years. (Intensive Care Nurse 1)

Several mentioned that their interest in the field outweighed the high workload and shift work. “It is that we are valued. In terms of salary, first and foremost. The right people need to understand what it means, the work we do.” (Intensive Care Nurse 8).

Although the participants appreciated the ICU leadership and felt heard when expressing concerns about resource cuts, they were worried about the broader trends in the Norwegian healthcare system, where resource reductions and a heightened focus on savings could potentially compromise patient care. *“*Yes, and there won’t be any hiring to cover illnesses. It will be interesting to see how things unfold as they need to save even more. I think it might lead some to retire earlier” (Intensive Care Nurse 4).

Additionally, they mentioned an increasing trend of being reassigned to other departments and wards due to staffing shortages or high patient loads in those areas. While the participants acknowledged the importance of assisting other units, they expressed concerns that such resource management could hinder their professional development. They feared that this could negatively affect their workday and potentially lead to burnout. “I would rather do something professional in the department and focus on what we are supposed to do here. But if it is critical and they need help, of course, we will help, that’s not what I mean. But having a system for it, I am not in favour of that. I think we will just wear ourselves out. (Intensive Care Nurse 6)

### Professional Engagement and Mastery

The participants emphasised the importance of the work environment’s mutual focus on care quality, proper training, continuous skill development, and task distribution based on competence and experience. They said that their ability to professional development contributed to increased mastery and motivation to continue in the profession. Several participants highlighted that working in an environment with such a strong interest in intensive care nursing was inspiring. They found it rewarding to work with severely and critically ill intensive care patients. “It’s the patient treatment, and making sure the patients are okay, in spite of the hard treatment that is given. And then to use what you have learned to basically do a good job with the patients” (Intensive Care Nurse 2).

Being able to help and support both patients and the patients’ relatives or next of kin in various crises, verified that they were doing an important job, which was motivating in itself.

I probably like following up with patients over a longer period and seeing them improve. From being very ill to getting better. I find that incredibly rewarding, and it’s very enjoyable when they start the rehabilitation phase and you get to work with their family and children, yes. . . Relatives too. (Intensive Care Nurse 7)

Effective teamwork, where all professions collaborated well for the benefit of the patient, was also highlighted as important and brought the intensive care nurses great joy in their daily work. Additionally, they emphasised the importance of developing together in a multidisciplinary environment, both among intensive care nurses and in collaboration with doctors. “We don’t need to talk much, everyone knows what needs to be done, and things get done. I think that works well.” (Intensive Care Nurse 6)

## Discussion

The aim of this study was to explore what motivates nurses to work in the ICU. We found that a work environment where everyone feels acknowledged and heard, and where the culture emphasises creating a safe and enjoyable workplace for all, is crucial for the nurses motivation to continue working in the ICU. These findings are consistent with the Self-Determination Theory (SDT) framework (Deci et al., 2017), which explains that employees’ performance and well-being are influenced by the type of motivation they have for their job activities. The framework also highlights what fosters high-quality, sustainable motivation in employees. When employees feel supported in their autonomy, it leads to greater satisfaction and thriving, along with collateral benefits for organisational effectiveness (Deci et al., 2017). The intensive care nurses in this study felt supported in their autonomy, and they enjoyed working in the ICU because there was an open culture for discussing challenging situations to provide the best possible care for the patient. They also highlighted the importance of proper training, continuous skill development, and task distribution based on competence and experience. Being engaged in professional development and trusted to perform tasks that matched their skills made them feel empowered. This empowerment allowed them to take initiative and feel in control of their work, which boosted their motivation and job satisfaction (Deci et al., 2017). The participants, however, stressed the importance of ensuring that no one was given responsibilities beyond their skills and capabilities. Other researchers have reported that intensive care nurses valued being resources for one another because it fostered a sense of security within the team (Høgbakk & Jakobsen, 2019). Professional and practical competence enhancement through scenario training, experience exchange, and professional development days were also highlighted as important for why the nurses wanted to work in the ICU. This finding is consistent with other studies that underscore the value of internal training and professional development, particularly in enhancing their skills and confidence in challenging situations (Alshahrani, 2020; Mlambo et al., 2021; Pandian et al., 2024). Making the intensive care nurses feel competent and confident in their abilities was also crucial for maintaining high levels of motivation and job satisfaction, as it enabled them to perform their duties effectively and feel accomplished in their roles (Deci et al., 2017). The participants in this study experienced that the management emphasised competence development and professional challenges, which was motivating. Unfortunately, this is not the case in all ICUs, there are workplaces where nurses do not always experience support to pursue further education and professional development (Høgbakk & Jakobsen, 2019).

The findings in this study also align with how the SDT motivation model for the workplace demonstrates that when employees receive support for their autonomy, they generally feel more connected to the organisation and perceive themselves as more effective (Deci et al., 2017). The participants in this study described that the interpersonal and professionally focussed work environment, which included giving and receiving feedback and recognition for their work, motivated them to work in the ICU. The importance of holding regular section meetings and providing opportunities for staff to influence their workday and processes in the ICU were also contributing to their motivation and are indicative of a work environment characterised by structural empowerment (Orlowska & Laguna, 2023). Such workplaces stand in positive contrast to workplaces where nurses do not feel valued and listened to by the top management, resulting in nurses wanting to leave their jobs (Eriksson et al., 2022). From an SDT perspective, leaders who enthusiastically communicate ideas and support employees in ways that strengthen them will better fulfil employees’ basic psychological needs for competence, autonomy, and relatedness. By acknowledging employees’ perspectives during discussions, offering choices on how to implement ideas, and avoiding pressuring behaviours and language, leaders can more effectively foster employees’ autonomous motivation (Deci et al., 2017). The participants in this study highlighted the need for active and continuous efforts to maintain a positive work environment. They could easily become dissatisfied, particularly if management decisions were made without their input and if they felt minimally involved. Other studies show that leaders who appear authentic and are concerned with safeguarding their employees’ interests through competence enhancement and development, are important factors for employees to thrive at work (Spence Laschinger & Fida, 2015; Wei et al., 2018) and for nurses wanting to work as nurses (Spence Laschinger & Fida, 2015). Interdisciplinary collaboration with doctors, where caring for the intensive care patient was their primary focus, was also highlighted as a positive contributing factor for their job satisfaction, also emphasised in other studies (Al Sabei et al., 2022; Høgbakk & Jakobsen, 2019). This stands in positive contrast to studies showing that lack of involvement and collaboration between intensive care nurses and doctors can be a negative factor in perceived job satisfaction (Georgiou et al., 2017; Hasanabadi et al., 2023). However, teamwork is stated as essential for both patient safety and providing a healthy work environment (Hasanabadi et al., 2023).

The participants in this study had varying opinions on what constitutes acceptable shift patterns. This aligns with other studies indicating that personal characteristics and circumstances influence perceptions of acceptable shift lengths, shift times, and overall shift patterns (Ejebu et al., 2021; Emmanuel et al., 2024). The participants also noted that the overall salary, including allowances for inconvenient working hours, had improved. They emphasised that good salary conditions are crucial for ensuring further recruitment, a point supported by another study (Abu Yahya et al., 2019). Although the participants generally experienced good staffing levels, they hinted at an unfortunate development where demands for savings led to reduced hiring during illness.

In summary, the findings align with the principles of SDT (Deci et al., 2017) by demonstrating how environmental factors in the ICU, such as professional support, effective leadership, and opportunities for skill development, fulfil the intrinsic needs of autonomy, competence, and relatedness. These factors contribute to intensive care nurses’ motivation and job satisfaction, explaining why they remain in their profession. Understanding these connections can help in designing interventions and policies that enhance the work environment and support the well-being of intensive care nurses.

### Strengths and Limitations

One of the strengths of this study is the qualitative design and the collection of in-depth data through individual semi-structured interviews. The interviews allowed the participants to openly and honestly share their experiences of the work environment in the ICU with an external researcher. Since the interviewer was a nurse herself, it made it easier for her to recognise similar situations and ask follow-up questions about how the participants experienced different situations, for example, when the participants shared their experiences of being moved to other departments and wards due to staffing shortages or high patient loads in those units. Another strength is the characteristics of the research team. The team was composed of nurses and researchers well-versed in the study topic, with varying levels of clinical and research experience, which complemented each other. It is also strengthening that the study’s information power is sufficient (Malterud et al., 2016). After conducting six interviews, it was assessed that the information power was achieved. Nevertheless, two additional interviews were conducted, showing that the content was consistent with the previous six. The participants shared many of the same perceptions of their experiences, contributing to the reliability of the data and indicating that it is not just one individual experience, but a shared experience among several.

A possible limitation of the study is that nurses who were uncertain about remaining in their positions were unlikely to volunteer for this study, given its focus on motivation for staying in this ICU. Another potential limitation is that we only included nurses from a single ICU, making the findings specific to this unit and not necessarily representative of what motivates nurses to continue working in ICUs more broadly. On the other hand, the findings may still be transferable to other ICU settings in larger hospitals and different countries, since some of the important factors for employees to thrive at work in this study are shown among employees in general (Wei et al., 2018), and they are in line with the theory of self-determination (Deci et al., 2017).

### Implications for Practice

The findings in this study have several practical implications. To ensure an adequate supply of intensive care nurses in the future (Lasater, 2024), we recommend focussing on professional development implemented in rotation shifts, salary and adequate staffing levels, workplace senior policy to prevent unwanted early retirement and make it more attractive for seniors to continue working longer, and strengthening the leadership relationships within the ICU. Regular feedback mechanisms and open-door policies can facilitate the latter. Additionally, it is important to create opportunities for staff to discuss and reflect on their daily work and involve intensive care nurses in decision-making processes that affect their workday. This may involve strategic workforce planning, recruitment drives, and retention strategies.

## Conclusion

This study found that a supportive and inclusive work environment, where colleagues look out for one another and prioritise professional development, greatly impacts intensive care nurses’ motivation to remain in their roles, and continue working in the ICU. Other essential factors for staying in the profession included having a trusting relationship with their immediate leader, responsive management that advocates for them, opportunities for discussions and reflections on their daily work, and involvement in decisions affecting their workday. Adequate staffing to ensure high-quality intensive care and to feel secure and competent in their roles was also highlighted as very important.
